# Exploring radiation-free scoliosis monitoring: systematic review and meta-analysis of non-ionizing methods

**DOI:** 10.1186/s12891-025-09034-8

**Published:** 2025-10-01

**Authors:** Martin Bertsch, Lucrezia Mulatero, Sina Salehpour, Mirko Kaiser, Volker M. Koch, Christoph J. Laux, William R. Taylor, Sasa Cukovic

**Affiliations:** 1 Laboratory for Movement Biomechanics, ETH Zurich, Zurich, Switzerland; 2 Biomedical Engineering Lab, Bern University of Applied Sciences, Biel, Switzerland; 3 Department of Physical Therapy and Rehabilitation Science, University of Maryland, Baltimore, USA; 4 University Spine Center Zurich, Balgrist University Hospital, University of Zurich, Zurich, Switzerland

**Keywords:** Scoliosis, Monitoring, Follow-up, Radiography, Ionizing radiation, Radiation-free, Meta-analysis, Systematic review

## Abstract

**Background:**

Idiopathic scoliosis is a three-dimensional spinal deformity that typically develops during childhood or adolescence but may affect individuals across the lifespan. Regular monitoring is often necessary to detect progression and assess treatment effectiveness. Radiography remains the clinical gold standard; however, repeated ionizing radiation exposure is associated with increased cancer risks, highlighting the need for reliable, non-invasive, and radiation-free assessment methods. This systematic review and meta-analysis evaluated the diagnostic accuracy and criterion validity of emerging radiation-free scoliosis monitoring techniques compared to radiographic standards.

**Methods:**

A comprehensive literature search across six databases (Cochrane, EMBASE, IEEE Xplore, PUBMED, Scopus, Web of Science) identified 56 eligible studies involving 4,774 patients diagnosed with idiopathic scoliosis (median number of patients per study: 38; range: 5 to 952, mean patient age: 15.2 years, female-to-male ratio: 3:1). Criterion validity was assessed by pooling Pearson correlation coefficients between radiographic and non-ionizing measurements. Measurement accuracy was assessed by pooling their mean absolute differences in Cobb angles. Additionally, sensitivity and specificity for detecting deformity progression were assessed. Statistical analyses employed multilevel linear mixed-effects models, introducing moderators to explain study heterogeneity.

**Results:**

Ultrasonography demonstrated the highest overall validity, consistently correlating strongly (r≈0.9) with radiographic Cobb angles. Surface topography also showed robust correlation (*r* > 0.8), although evidence remains insufficient for patients with higher body mass indices or more severe spinal curvatures for both methods. Magnetic resonance imaging exhibited a very strong correlation (*r* = 0.93) with radiographic measurements; however, correlation varied significantly depending on patient positioning. Upright MRI provided more consistent results compared to supine positioning.

**Conclusions:**

Ultrasonography and surface topography represent promising radiation-free alternatives that could significantly reduce radiographic assessment frequency, minimizing radiation exposure, particularly in suitable patient groups. While MRI also demonstrates excellent validity, its broader clinical applicability remains constrained by substantial costs, limited availability, and extended examination durations. Although these non-ionizing modalities are not yet viable replacements for routine radiography, their demonstrated validity and accuracy supports their potential as complementary technologies, particularly for screening or supplementary monitoring of scoliosis.

**Supplementary Information:**

The online version contains supplementary material available at 10.1186/s12891-025-09034-8.

## Background

Scoliosis is a condition characterized by a three-dimensional deformation of the spine, mainly leading to deviations in the coronal plane and often axial rotation. Untreated and progressing spine deformities are associated with back pain, cardiopulmonary symptoms, and an increased mortality rate [1]. Adolescent idiopathic scoliosis (AIS) is the most common spinal disorder in children and adolescents, comprising over 85% of all scoliosis cases and affecting 2–4% of individuals aged six to fourteen [2, 3]. To quantify the degree of lateral curvature, the Cobb angle (CA) is obtained from an anteroposterior radiograph. The CA is defined as the angle between the upper endplate of the most tilted vertebra cranially, and the lower endplate of the most tilted vertebra caudally around the apex of the curve, where a CA of more than 10° is considered pathological [4–8]. Also, scoliosis is classified into three severity levels “mild” (CA of less than 25°), “moderate” (between 25–40°), and “severe” (more than 40°) [9, 10]. Due to the risk of deformity progression, regular monitoring is necessary, particularly in individuals where skeletal maturity has not yet been reached, substantial growth potential remains, or other relevant risk factors are present. In such cases, radiographic imaging is typically performed every three to six months to assess deformity progression and evaluate treatment effects [11].

The most widely used clinical method, and currently considered the gold standard for monitoring scoliosis, is X-ray imaging, which is ubiquitously available throughout hospitals and clinics. It allows direct assessment of the vertebral column and potential spinal structural pathologies. Over the course of several years, a patient with scoliosis undergoing treatment may receive 10 to 25 spinal X-rays, primarily due to the need for regular monitoring of curve progression, assessing the effectiveness of interventions such as bracing, physiotherapeutic scoliosis-specific exercises, or observation, and ensuring proper brace fit and adjustment. In individuals diagnosed at an earlier age, the cumulative number of radiographs can be even higher, when substantial growth potential and a longer treatment duration are expected [12]. Cumulative exposure due to clinical imaging for scoliosis monitoring is known to increase the carcinogenic risk, and therefore, cancer-related mortality [13–20]. For example, Simony et al. provided evidence that the incidence of cancer among individuals with scoliosis is 5 times greater than that of the general aged-matched population [16]. Ronckers et al. followed up 5′513 female subjects who were monitored for scoliosis and reported that, on average, subjects experienced 23 radiographs during their treatment and follow-up [14]. Cancer-related mortality among these patients was 1.46 times higher compared to the general population. Similarly, Knott et al. reported that the risk of breast cancer mortality was found to be 1.4 times greater for scoliosis patients who had undergone 25 to 49 X-rays with breast exposure compared to those who had received fewer than 25 X-rays [13]. Women who had undergone more than 50 X-rays had a 2.7 times increase in risk of breast cancer-related mortality [20]. This overwhelming evidence indicates a clear correspondence between the number of X-rays and the increase in risk of mortality resulting from cancer, if the thoracic soft tissue is irradiated [13].

Given this important shortcoming of radiograph-based scoliosis monitoring, several non-ionizing techniques have been proposed as potential alternatives to radiography [21–27]. These techniques include surface topography (ST), ultrasonography (US), magnetic resonance imaging (MRI), and photogrammetry (PG). In addition, some other devices and approaches such as the Spinal Mouse [28], inclinometers [29], and digital computer vision-based techniques [23, 30] have all been Suggested. In some studies, the potential reduction in ionizing radiation from replacing X-ray follow-up with ST-based assessments was estimated at 30% to 71%, averaging 47% [27, 31–34]. Many studies have investigated the validity of these non-ionizing techniques [35–38]. For example, a strong Pearson correlation of 0.82 was reported between radiographic CA and ultrasound-derived spine angle measurements in a cohort of over 900 scoliosis patients [39]. These figures were corroborated by several other studies involving US in assessing scoliosis [37, 40, 41]. Moderate to strong correlations have also been reported between ST and radiographic examinations [42–44]. Latest MRI studies involving AIS patients also indicate an excellent correlation of up to 0.98 with radiographic examination [45]. However, the validity was often only assessed in cross-sectional studies rather than longitudinal follow-up studies. Comprehensive prospective long-term studies would be necessary to compare and validate radiation-free scoliosis assessment techniques against the gold standard to disrupt the current clinical practice regarding monitoring of scoliosis patients. Given the lack of such studies, a thorough comparison of published articles that considers different parameters and technique advantages/disadvantages is clearly necessary to achieve this objective.

In this work, relevant studies in the literature that assess techniques not involving ionizing radiation for scoliosis monitoring were systematically reviewed. Their validity in relation to the gold standard radiological measurements was assessed in a meta-analysis. Overall, this work aims to reveal novel techniques and their validity and accuracy in assessing AIS towards reducing the dependence on X-ray imaging.

## Materials and methods

### Search strategy

From the research question “In the context of scoliosis monitoring, what is the current validity and accuracy of radiation-free scoliosis assessment methods compared to gold-standard radiographic examination?” the PICO scheme was developed (population (P): patients with idiopathic scoliosis; intervention (I): radiation-free scoliosis assessment (index test); comparison (C): standing plain film radiography (reference standard); outcome (O): validity and accuracy of index tests compared to the reference standard). An initial inclusive single-line search query was constructed incorporating three conceptual blocks (the target condition (scoliosis), terms for radiation-free assessment methods (approaches and devices), and terms related to monitoring). The preliminary query was tested in EMBASE. Retrieved articles were screened by relevance, and additional synonyms and related terms were incorporated to improve sensitivity. To increase specificity, a fourth conceptual block targeting the reference standard (terms related to the reference standard) was added. Common terms from irrelevant articles were identified and used to construct an exclusion block. The final search query, detailed in Table 1, was adapted for use across six scientific databases to ensure comprehensive coverage: EMBASE, PUBMED, Cochrane Library, IEEE Xplore, Scopus, and Web of Science. The query structure combined columns 1–4 using the Boolean operator “AND”, while employing the “NOT” operator to exclude studies outside the intended scope (last column). The search focused on titles and abstracts and included studies published from the year 2000 through August 2024.

**Table 1:** The review search query. Columns 1–4 were combined with Boolean “AND”, and column 5 with “NOT”, while rows were connected with Boolean “OR”. Search fields: title and abstract. Note: The asterisk (*) was used as a truncation symbol to include all terms that begin with the root word (e.g., “scolio*” retrieves “scoliosis,” “scoliotic,” etc.)

Target condition	Reference standard and variations	Monitoring and synonyms	Index test: approaches and devices	Excluded terms
scolio*	cobb ionization-free ionizing noninvasive non-invasive nonionizing non-ionizing non-radiographic radiation radiation-free radiograph* x-ray	analys* assess* check* diagnos* evaluat* examin* follow* measur* monitor* observ* progress* record* screen* stud* surveil* track*	alternative imaging inclinometer intelligen* learning motion MRI network* optical photogrammetry rasterstereograph* resonance scan* scoliometer spinal Mouse surface stereoscop* substitut* surrogate thermograph* topograph* ultrasonography ultrasound	animal cadaver* mice pregnan* spinal fusion surgery tethering

### Selection process

Retrieved articles were imported into EPPI Reviewer 6 [46], and duplicates were automatically removed. All articles were examined using a pre-defined stringent set of eligibility criteria towards non-ionizing methods that were investigated or applied for scoliosis monitoring. Three independent reviewers screened papers for inclusion, first based on titles and keywords, and then based on abstracts. Each potential publication was then full text screened to confirm eligibility towards addressing the research question. Furthermore, the references of all included publications were screened for further eligible studies. Eligibility criteria were as follows:

- Inclusion criteria:
  1. Studies that use or propose a radiation-free modality for scoliosis follow-up.
  2. Studies that included participants with a clinical or radiographic diagnosis of idiopathic scoliosis, with a focus on adolescent idiopathic scoliosis.
  3. Metrics of criterion-based validity and/or (diagnostic) accuracy of the modality were evaluated and reported.
  4. The radiation-free modality was directly compared to radiographic examinations (standing plain films in coronal and/or sagittal plane).
- Exclusion criteria:
  1. Studies related to population screening, without specifically addressing monitoring of scoliosis.
  2. Studies examining cohorts who had undergone corrective surgery for scoliosis (as these patients do not qualify for most radiation-free monitoring techniques).
  3. Case studies; studies related to pregnant women, as well as all types of scoliosis other than idiopathic.
  4. Studies involving animals or cadavers.
  5. Studies in the form of abstracts, reviews, or book chapters.
  6. Articles not written in the English language.

### Quality assessment

The risk of bias and applicability concerns of each included study were evaluated using the Quality Assessment of Diagnostic Accuracy Studies-2 (QUADAS-2) tool [47]. This tool assesses four key domains: patient selection, index test, reference standard, and flow and timing. In cases of discrepancies, reviewers reached a consensus through discussion. Each study was then assigned a risk level of “high,”, “moderate”, “low,” or “unclear” for bias and applicability concerns. A detailed description of how the quality was judged can be found in Supplementary Table 3. As recommended, all relevant evidence was reviewed regardless of the quality assessment, and no quality score was computed to weight the studies [47]. However, possible limitations indicated by the quality assessment were considered when evaluating the applicability of radiation-free monitoring techniques.

### Data collection

Extracted information from the considered studies included author(s), publication year, cohort characteristics (scoliotic and healthy volunteers, number of subjects, sex, and age), imaging modalities used, device names, measured parameters for comparison with X-rays, follow-up time, radiation reduction, differences in CA between radiation-free modality and radiograph, correlation coefficients, linear regression analysis results, and sensitivity and specificity for detecting scoliosis progression. Three authors were involved in the data extraction process.

Regarding the validity of methods, attention was given to (a) reported metrics that corresponded to or were compared with the radiographic CA, and (b) modality-specific indicators of deformity progression, where the radiological equivalent would be an increase in the primary CA of more than 5°.

### Data synthesis and statistical analysis

Scoliosis assessment techniques were firstly categorized based on their technological methodology (e.g., ultrasound imaging) or similarity of the examination method (e.g., palpation and location of spinous processes). Subsequently, all the categorized methods were scrutinized for criterion validity and (diagnostic) accuracy metrics.

#### Measures for validity and accuracy assessment

Criterion validity and (diagnostic) accuracy were assessed by comparing how closely measurements from radiation-free techniques aligned with the reference standard, i.e., the CA derived from radiographs. The observed effect sizes ($y$) from primary studies were:

- Pearson correlation coefficients, measuring the correlation between radiographically derived CAs and those derived via the radiation-free method.
- Mean absolute differences (MAD) between these two, including measures of uncertainty (standard deviation, standard error, confidence intervals, or variance).
- Sensitivity and specificity in detecting deformity progression between monitoring visits using a radiation-free modality, with progression defined as an increase in radiographically measured Cobb angle of ≥ 5°.

The number of patients, $n$, of each study was used to weight the contribution of each effect size. The criteria for interpreting the correlation were: *r* > 0.9 was considered very strong, > 0.8 and 0.9 strong, > 0.6 and 0.8 moderate, between 0.4 and 0.6 low, and *r* < 0.4 very low.

#### Meta-analysis: statistical models

Statistical analyses were conducted using the R Statistical Software (version 4.2.3, R Core Team 2023) and the *metafor* package [48]. Each Pearson correlation effect size, $r$, was converted to Fisher’s $z$ scale (Eq. 1), and the corresponding sampling variance computed from the sample size, $n$ (Eq. 2). After pooling, the combined effects and their confidence intervals were transformed back to correlation units (Eq. 3) [49]. For effect sizes represented as MAD, variances were approximated using the sample standard deviation, $SD$, and the sample size, $n$ (Eq. 4). If raw patient-level data were available in the publications, Pearson correlation coefficients and mean absolute differences were calculated directly to confirm the reported effect sizes or to compute them when they were not explicitly provided. Sensitivity (Se) and specificity (Sp) effect sizes were logit-transformed (Eq. 5), while their standard errors, $SE$, were calculated from the 95% confidence intervals (CI) (Eq. 6) and adjusted for the logit-transformation (Eq. 7) [50]. After pooling, effect sizes and confidence intervals were transformed back using the inverse logit function (Eq. 8) [50]. If the 2 × 2 confusion matrix was reported, sensitivity and specificity were calculated directly to confirm the reported effect sizes or to compute them when they were not explicitly provided.

1 $$\begin{array}{c}z=\frac{1}{2}\text{ln}\left(\frac{1+r}{1-r}\right)\end{array}$$

2 $$\begin{array}{c}{v}_{z}=\frac{1}{n-3}\end{array}$$

3 $$\begin{array}{c}r=\frac{{e}^{2z}-1}{{e}^{2z}+1}\end{array}$$

4 $$\begin{array}{c}{v}_{MD}=\frac{{SD}^{2}}{n}\end{array}$$

5 $$\begin{array}{c}{\text{l}}{\text{o}}{\text{g}}{\text{i}}{\text{t}}\left({\text{Se}}\right)=\text{log}\left(\frac{\text{Se}}{1-{\text{Se}}}\right)\end{array}$$

6 $$\begin{array}{c}SE=\frac{C{I}_{high}-C{I}_{low}}{2\times 1.96}\end{array}$$

7 $$\begin{array}{c}S{E}_{logit}=\frac{SE}{{\text{logit}}\left({\text{Se}}\right)\left(1-{\text{logit}}\left({\text{Se}}\right)\right)}\end{array}$$

8 $$\begin{array}{c}Se=\frac{{e}^{{\text{logit}}\left(Se\right)}}{1+{e}^{{\text{logit}}\left(Se\right)}}\end{array}$$

To combine effect sizes, multilevel linear mixed-effects models were used to accommodate the heterogeneity across studies [51]. Studies were considered independent, and a random effect was assigned to each effect size. When multiple effect sizes were extracted from the same study or from studies from the same authors, non-independence was accounted for by including a random effect for these studies [48, 52]. In this approach, the observed effect size ${y}_{ij}$ from each ${i}^{th}$ independent study (with possibly multiple effect sizes per study, $j=1\dots J$) was assumed to represent a normally distributed estimate of the true effect ${\theta }_{ij}$ with sampling variances, ${v}_{ij}$, which were assumed to be known (Eq. 9). The true effects, ${\theta }_{ij}$, were assumed to be normally distributed with a mean true effect, $\mu$, and variance, ${\tau }^{2}$ (Eq. 10). The ${j}^{th}$ effect size from the ${i}^{th}$ study, ${y}_{ij}$, could therefore be described by the mean true effect, $\mu$, a random effect for the between-study variability, ${u}_{i}$, (i.e. heterogeneity across studies), a random effect for within-study variability, ${v}_{ij}$, and a sampling error, ${\varepsilon }_{ij}$ (Eq. 11). The combined effect size, $\widehat{\mu }$, and variance, ${\tau }^{2}$, were then estimated using Eq. 12 [48].

9 $$\begin{array}{c}{y}_{ij}\sim N\left({\uptheta }_{ij},{v}_{ij}\right)\end{array}$$

10 $$\begin{array}{c}{\uptheta }_{ij}\sim N\left(\mu ,{\tau }^{2}\right)\end{array}$$

11 $$\begin{aligned} {y}_{ij}=&\mu +{u}_{i}+{v}_{ij}+{e}_{ij}\,{\text{w}}{\text{i}}{\text{t}}{\text{h}}\,{u}_{i}\sim N\left(0,{\tau }_{\text{study}}^{2}\right), \\&{v}_{ij}\sim N\left(0,{\tau }_{\text{within}}^{2}\right), {\varepsilon }_{ij}\sim N\left(0,{v}_{ij}\right)\end{aligned}$$

12 $$\begin{array}{c}\widehat{\mu }=\frac{\sum {w}_{ij}{y}_{ij}}{\sum {w}_{ij}}\hspace{1em}{\text{w}}{\text{i}}{\text{t}}{\text{h}}\hspace{1em}{w}_{ij}=\frac{1}{{\tau }_{\text{study}}^{2}+{\tau }_{\text{within}}^{2}+{v}_{ij}}\end{array}$$

To obtain an approximately unbiased estimation of the variance between and within studies, the restricted maximum likelihood estimator was used [48, 53]. Moderators that might explain heterogeneity across studies were included as fixed effects in subgroup analyses. Categorical moderators included the spinal region (thoracic, lumbar, or thoracolumbar), the evaluation approach within the same technique, and the device used for scoliosis assessment. A minimum requirement of three effect sizes was defined to include a moderating group in the analysis. Each moderator was analyzed in a separate model to explore the individual effects of moderator variables and to account for limited data. The model was then further described by the moderator variable ${x}_{i}$ for the ${i}^{th}$ study, where ${\beta }_{0}$ and ${\beta }_{1}$ represented the intercept and coefficient for the moderator, respectively (Eq. 13) [48].

13 $$\begin{array}{c}{y}_{ij}={\beta }_{0}+{\beta }_{1}{x}_{i}+{u}_{i}+{v}_{ij}+{e}_{ij}\end{array}$$

As a result, correlation estimates were calculated when Cobb angle-comparable metrics from a radiation-free scoliosis assessment method were compared with the radiograph-derived CA in the coronal plane. When available, correlation estimates were also calculated in the sagittal plane between thoracic kyphosis and lumbar lordosis obtained from radiographs and radiation-free methods.

## Results

### Article selection

**Fig. 1:**
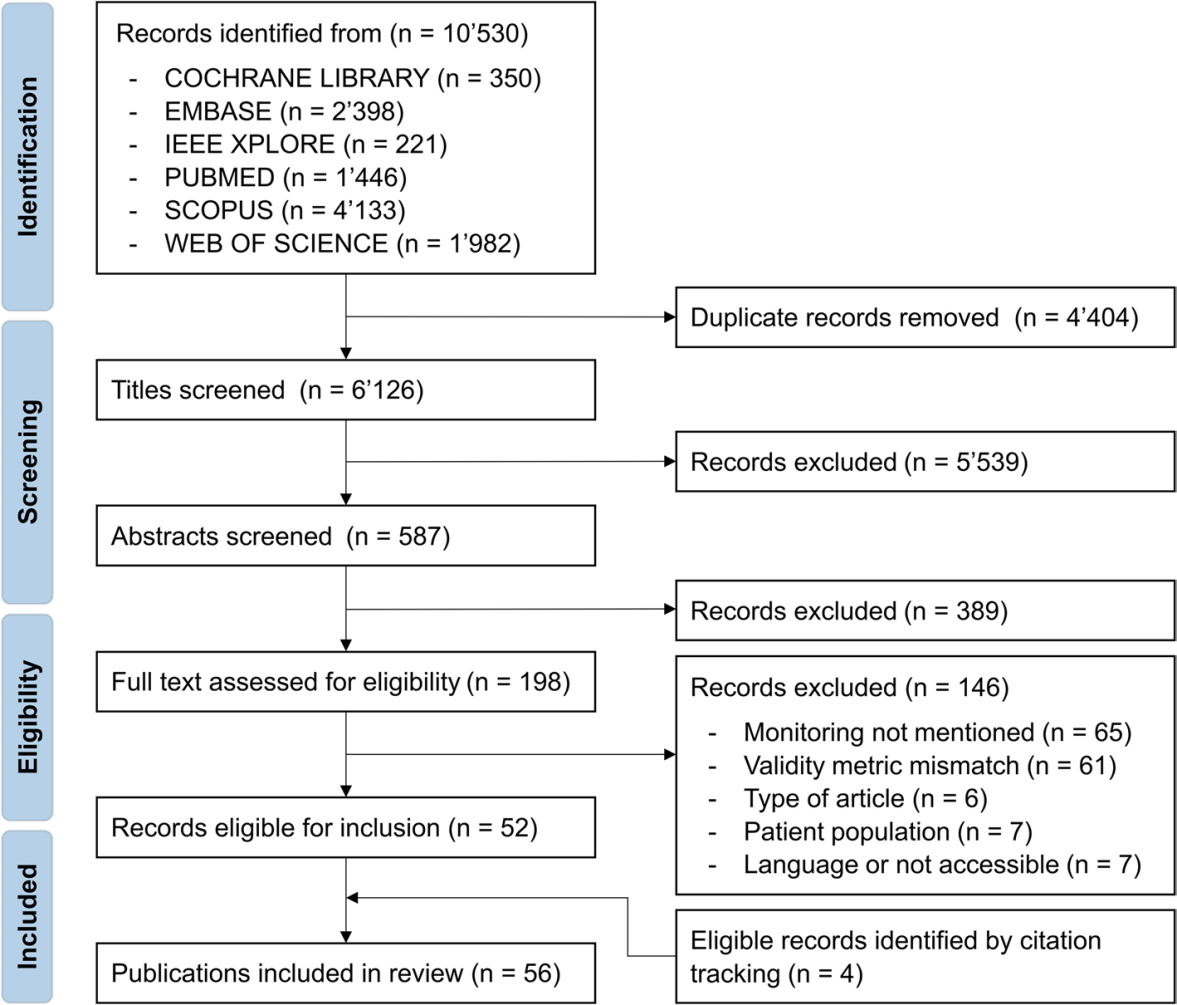
PRISMA flow diagram for systematic reviews with included searches of databases and registers. The total number of included publications in this systematic review was 56

This systematic search initially identified 10′530 records, of which 6′126 remained after eliminating duplicates (Fig. 1). These were then screened based on their titles and abstracts, leading to the exclusion of 5′928 records. Of the remaining 198 papers, 146 were removed after full-text review, and 4 included from citation tracking, leaving a total of 56 studies included in this systematic review. The references of these 56 included studies are marked in bold in the bibliography.

### Study characteristics and population demographics

This review included only studies that utilized or proposed a radiation-free modality for the follow-up monitoring of scoliosis. Of the 56 studies reviewed, 15 were longitudinal monitoring studies. The largest proportion of the studies included were prospective or retrospective cross-sectional studies (*n* = 41).

A total of 4′774 patients diagnosed with idiopathic scoliosis were included in the review (Supplementary Fig. 2, Supplementary Table 2). The median number of patients per study was 38, with a range from 5 to 952. From the available gender information, female patients (3′208) considerably outnumbered male patients (1′029) with a ratio of 3:1, while the mean age of all individuals was 15.2 years.

### Quality assessment

**Fig. 2:**
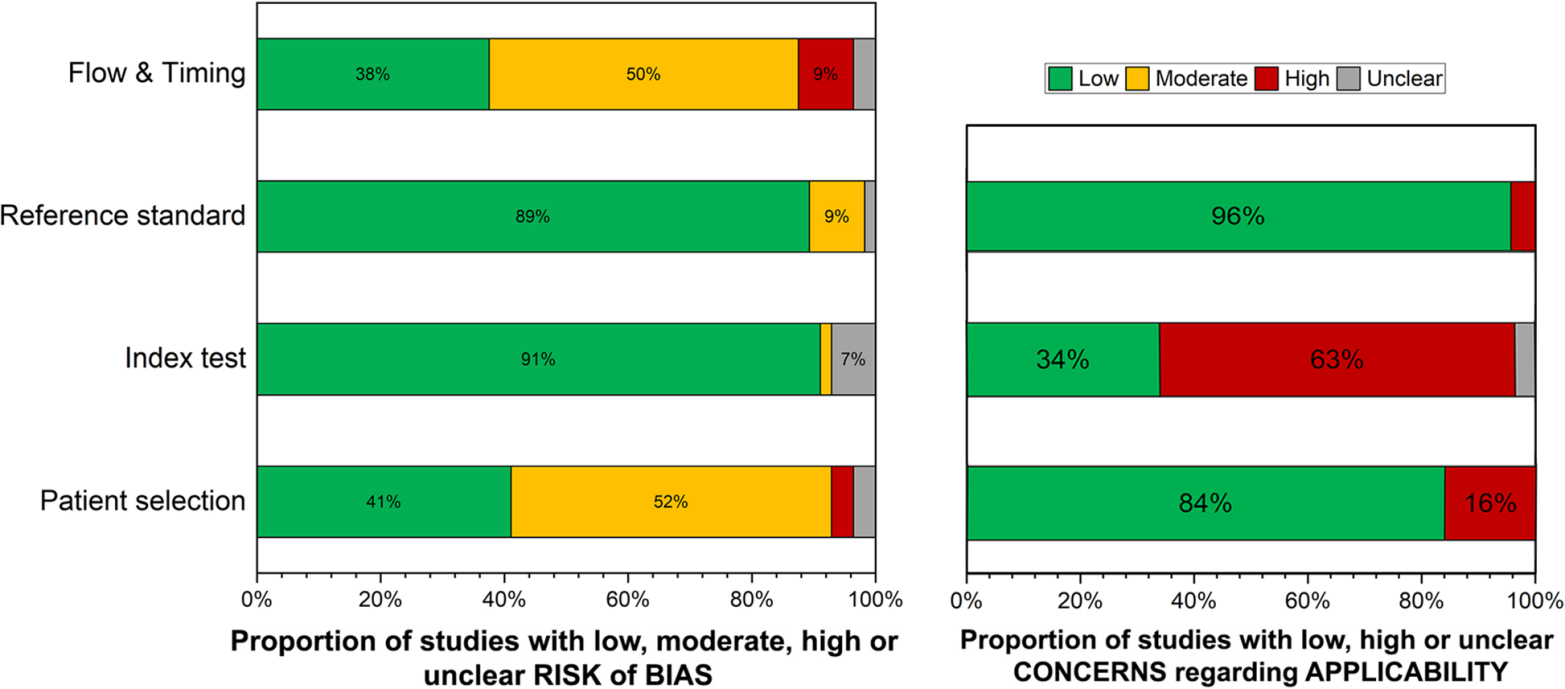
Quality assessment of included publications according to QUADAS-2. Left: risk of bias; right: concern of applicability

The QUADAS-2 quality assessment revealed considerable bias and applicability concerns in certain domains (Fig. 2, Supplementary Table 4). In the “Index Test” category, strong applicability concerns were noted in the majority of studies, affecting 35 out of 56. For “Patient Selection” and “Reference Standard” categories, most studies demonstrated low applicability concerns (47 and 55 studies, respectively).

When assessing bias, the “Flow & Timing” and “Patient Selection” categories revealed the most instances of moderate, high, or unclear risk outcomes (34 and 33 publications respectively). Conversely, the majority of publications (51 and 50 studies, respectively) were appraised as low risk for bias in both the “Index Test” and “Reference Standard” categories.

### Categorization of radiation-free scoliosis monitoring techniques

**Fig. 3:**
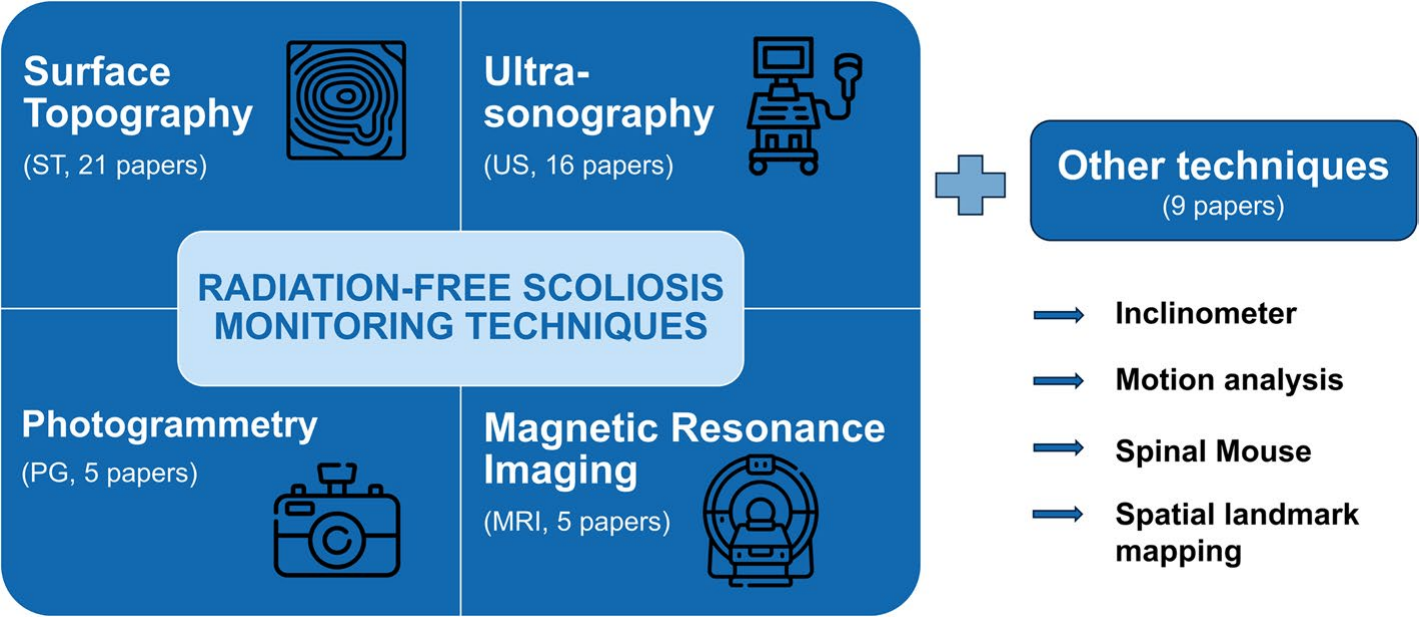
The main radiation-free assessment techniques used/tested or proposed for scoliosis monitoring identified in this systematic review. These are: surface topography (ST), ultrasonography (US), photogrammetry (PG), magnetic resonance imaging (MRI), and spatial landmark mapping, Spinal Mouse, inclinometer, motion analysis, summarized as “other techniques”

This review resulted in five primary radiation-free scoliosis monitoring technique categories. A brief description of the different techniques and their categorization can be found in Supplementary Table 1. These categories included surface topography (ST, 21 studies, $n=1{\prime}526$), ultrasonography (US, 16 studies, $n=2{\prime}059$), magnetic resonance imaging (MRI, 5 studies, $n=190$), photogrammetry (PG, 5 studies, $n=587$), and “other techniques” (9 studies, $n=412$) (Fig. 3). The latter category comprises inclinometers or scoliometers, and devices to locate and map spinous processes and possibly other landmarks on the back surface, be it with the help of a Spinal Mouse, a motion capture system or magnetic spine mapping. All these techniques have been used for, or were proposed for, the follow-up of scoliosis, specifically targeting the reduction of ionizing radiation during monitoring.

### Validity and accuracy of radiation-free scoliosis monitoring techniques

#### Surface topography

**Fig. 4:**
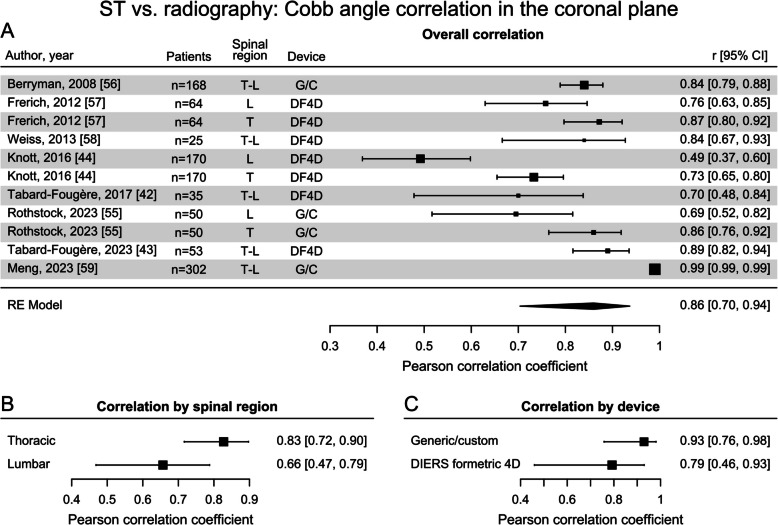
Forest plots displaying the Pearson correlation coefficients between surface topography (ST) Cobb angle estimates and radiographically-derived angles in the coronal plane: **A** overall correlation (spinal regions: lumbar (L), thoracic (T), combined thoracic and lumbar regions (T-L); devices: DIERS formetric 4D (DF4D), non-certified generic and custom (G/C); **B** correlation by spinal region; **C** correlation by device [42–44, 55–59]

The measured parameters and reported metrics in the ST category were highly heterogenous (Supplementary Table 5) [36]. The pooled CA correlation in the coronal plane obtained from the mixed effect meta-analysis with values from 8 studies was strong ($r=0.86$, Fig. 4A). Moderators accounting for variance from different spinal regions demonstrated a strong overall correlation ($r=0.83$) in the thoracic spine region, while the lumbar spine region exhibited only a moderate correlation ($r=0.66$) (Fig. 4B**)**. For the device moderator, the only commercially available and certified product for optical assessment of the spine, “DIERS formetric 4D”, showed a moderate correlation of $r=0.79$, while assessment based on generic and non-certified 3D scanners or self-developed systems demonstrated a strong correlation of $r=0.93$ with CAs assessed radiographically (Fig. 4C). The pooled overall MAD (reported in only 4 studies [42, 43, 54, 55]) was 5.5° (95% CI: [4.5°, 6.4°]).

**Fig. 5:**
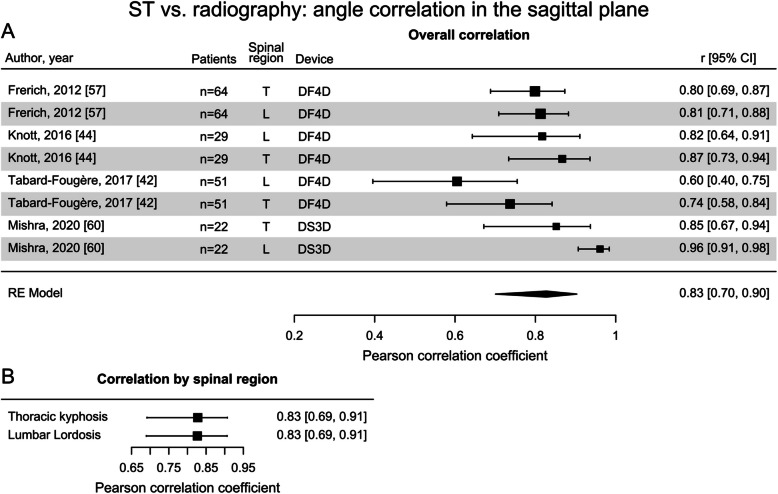
Forest plots displaying the Pearson correlation coefficients between surface topography (ST) angle estimates and radiographically-derived angles in the sagittal plane: **A** overall correlation (spinal regions: lumbar (L), thoracic (T); devices: DIERS formetric 4D (DF4D), DIERS Statico 3D (DS3D)); **B** correlation by spinal region [42, 44, 57, 60]

In the sagittal plane, the overall angle correlation (reported in only 4 studies) pooled in a meta-analysis was similarly strong as in the coronal plane ($r=0.83$, Fig. 5A). Similarly strong correlations for both thoracic kyphosis and lumbar lordosis were found ($r=0.83$, Fig. 5B). The number of effect sizes obtained were insufficient to introduce a moderator accounting for device differences in the sagittal plane.

Six studies [9, 22, 27, 33, 61, 62] reported the diagnostic accuracy of identifying deformity progression using ST (change in CA of more than 5°). The pooled sensitivity was 79% (95% CI: [69%, 87%]), while the pooled specificity was 61% (95% CI: [47%, 74%]).

#### Ultrasonography

**Fig. 6:**
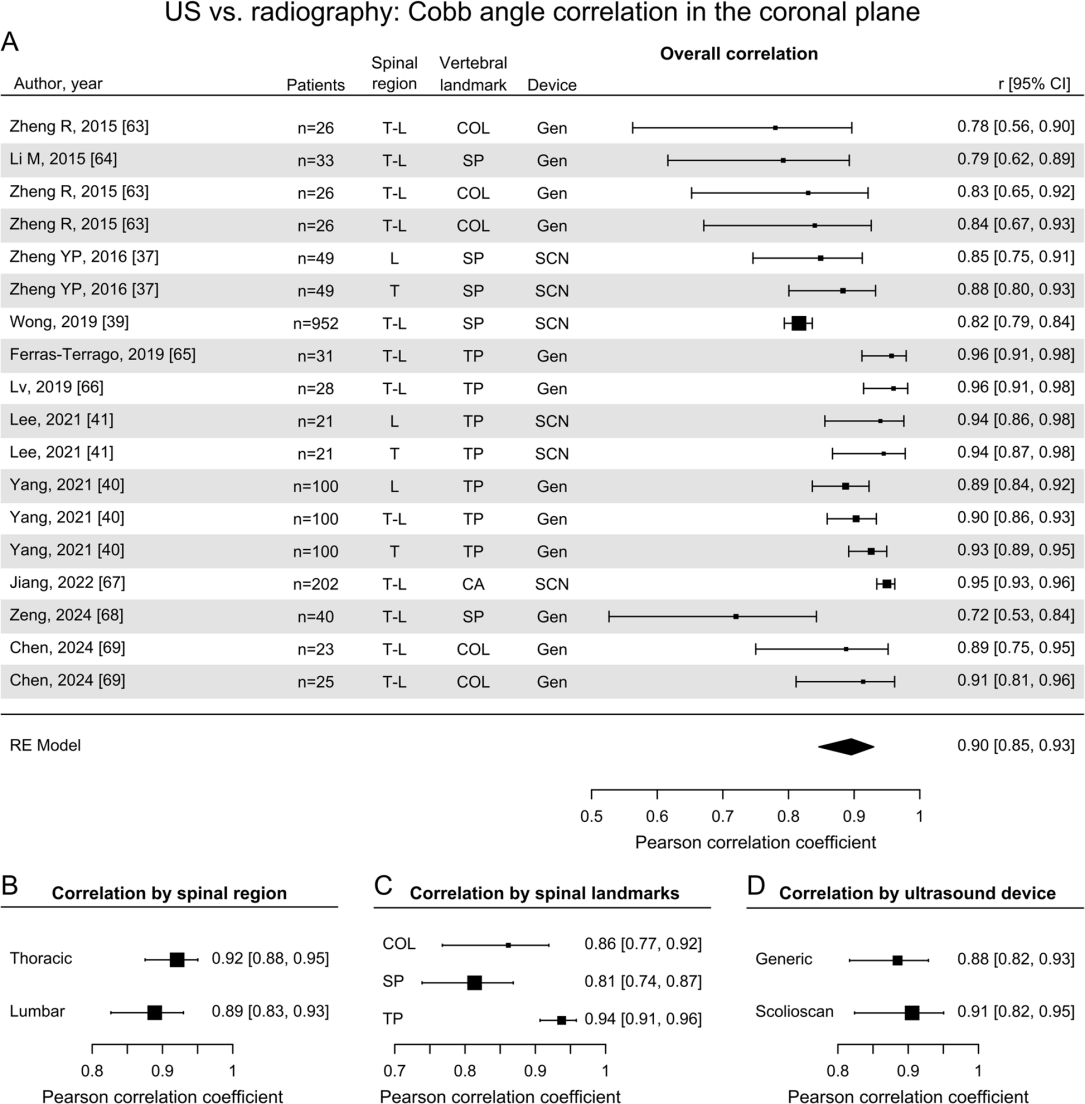
Forest plots displaying the Pearson correlation coefficients between Cobb angles obtained from ultrasonography (US) and radiography. Cobb angle correlations are shown **A** in the coronal plane (spinal regions: lumbar (L), thoracic (T), combined thoracic and lumbar regions (T-L); devices: Scolioscan (SCN), generic ultrasound probe (Gen)); **B** according to the spinal region; **C** according to the vertebral landmarks used to determine the angle (transverse process (TP), spinous process (SPA), center of lamina (COL)), and **D** according to the ultrasound device used [37, 39, 40, 40, 63–69]

Compared to ST, the measured parameters and reported metrics were more homogenous for the US category (Supplementary Table 6). The meta-analysis, based on 11 studies, revealed a strong overall correlation ($r=0.90$) between US-derived CAs and radiographically derived CAs (Fig. 6A). The strong correlations were consistent for both the thoracic and lumbar spine regions ($r=0.92$ and $r=0.89$, respectively) (Fig. 6B). Close examination of different vertebral landmarks to measure deformity angles revealed that the use of transverse processes exhibited the most robust and strongest correlation ($r=0.94$) with radiographic CA, whereas use of spinous processes and laminae produced less strong correlations ($r=0.81$ and $r=0.86$, Fig. 6C). Considering variability introduced by different devices as a fixed effect revealed similarly strong correlations between “Scolioscan” (application-specific) and generic ultrasound probes ($r=0.91$ and $r=0.88$, Fig. 6D).

**Fig. 7:**
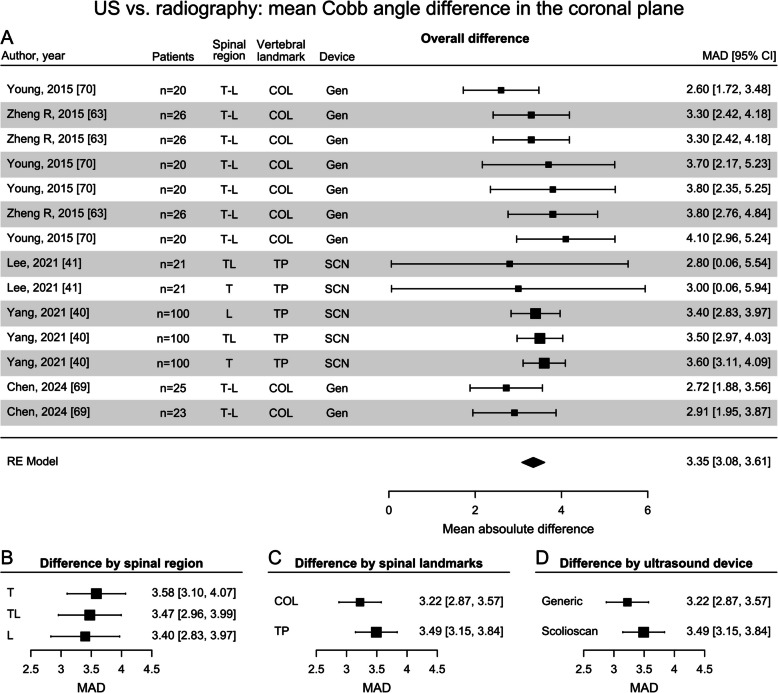
Forest plots displaying the mean absolute difference (MAD) between Cobb angles obtained from US and from the reference standard. Cobb angle differences are shown **A** in the coronal plane (spinal regions: lumbar (L), thoracic (T), combined thoracic and lumbar regions (T-L); devices: Scolioscan (SCN), generic ultrasound probe (Gen)); **B** according to the vertebral landmark used to determine the angle (transverse process (TP), spinous processes (SP), center of lamina (COL)); **C** according to spinal region, and **D** according to the ultrasound device used [40, 41, 63, 69, 70]

These findings were corroborated by the MAD analysis reported by 5 different studies, indicating an overall MAD between US Cobb angle and radiographic CA of 3.4° (Fig. 7A). The use of different vertebral landmarks resulted in comparable differences for TP (3.5°) and for laminae (3.2°, Fig. 7C). In terms of spinal regions, the lowest difference was observed for the lumbar region (3.4°), and while the thoracic and thoracolumbar regions showed slightly higher MADs (3.6° and 3.5° respectively) (Fig. 7B). Differences according to device were also comparable, with a MAD of 3.2° for evaluation based on generic ultrasound transducers, and 3.5° for the application-specific Scolioscan (Fig. 7D).

Only two studies [70, 71] within the US category reported the diagnostic accuracy of ultrasonography in identifying scoliosis progression, revealing a combined sensitivity of 89% (95% CI: [70%, 96%]), and a specificity of 93% (95% CI: [87%, 96%]). Also, only two studies [72, 73] investigated the angle correlations in the sagittal plane, with a moderate combined Pearson correlation ($r=0.80, 95 \% \text{CI}:\left[0.78, 0.83\right]$), whereas the MAD was only reported by the latter, which was on average 6.2° (95% CI: [5.9°, 6.6°]).

#### Photogrammetry

The pooled correlation (with effect sizes from 3 studies [74–76]) between radiographic CA and photogrammetry metrics were moderate ($r=0.68, 95 \% \text{CI}:\left[0.26, 0.88\right]$). Only one study [77] provided data on the MAD between radiographic CA and the CA estimated by computerized photogrammetry, where the average difference was 4.1° (95% CI: [2.3°, 6.0°]; thoracic: 2.9°, 95% CI: [1.6°,4.3°], and lumbar: 5.1°, 95% CI: [1.7°,8.6°]). The sensitivity and specificity combined from two studies [74, 78] was 84% (95% CI: [36%, 98%]) and 84% (95% CI: [74%, 91%]), respectively (Supplementary Table 7).

#### Magnetic resonance imaging

The Pearson correlation between MRI-derived CA and radiographically-obtained coronal CA pooled from 4 studies [45, 79–81] was very strong ($r=0.93, 95 \% CI:[\text{0.79,0.98}]$). An insufficient number of MADs with corresponding measures of uncertainty were gathered to allow meta-analytical pooling. The reported mean differences between MRI and radiographic CA measurements ranged from 0.6° to 5°. In the sagittal plane, strong correlations for thoracic kyphosis and low correlations for lumbar lordosis ($r=0.82$ and $r=0.55$) were reported by one study [81] (Supplementary Table 8).

#### Other techniques

##### Spatial landmark mapping

Spatial landmark mapping refers to palpating and mapping the spinous processes and possibly other landmarks on the back surface, e.g. using a fingertip sensor (Ortelius800 device [82, 83]) or a pointer stick (ZEBRIS system [84]). The pooled Pearson correlation between coronal radiographic spinal angles and those obtained from mapped landmarks (reported by 4 studies [82–85]) was strong ($r=0.85, 95 \% CI:[0.81, 0.89]$). The MAD pooled from three studies [82–84] was 5.1° (95% CI: [3.8°, 6.3°]). In the sagittal plane, the combined correlation for thoracic kyphosis from only 2 studies [82, 84] was strong ($r=0.86, 95\% CI:[0.81, 0.90]$), whereas the MAD was 5.4° (95% CI: [4.6°, 6.1°]).

##### Motion analysis

One study [86] reported low correlations between radiographically-obtained CA and those determined using a retroreflective marker-based optical motion tracking system. Another study [87] using gait analysis based on inertial measurement units and machine learning reported a sensitivity of 92% for detecting deformity progression. However, their models were evaluated without employing a separate unseen test set, which limits the assessment of generalization performance.

##### Scoliometer/inclinometer

The combined correlation between scoliometer angle of trunk rotation measurements during forward bending and coronal radiographic CA from 3 studies [88–90] was moderate ($r=0.64, 95 \% CI:[0.51, 0.74]$). In the sagittal plane, the correlation for TKA reported by one study [29] was moderate (*r* = 0.74), and negligible for LLA (*r* = 0.31) during upright standing.

##### Spinal mouse

The linear correlation between radiographic CA and Spinal Mouse coronal plane curvature was found to be moderate (*r* = 0.69 to 0.88), with mean differences of 3.1° to 4.4° [28], pointing towards an underestimation of the CA by the Spinal Mouse. The agreement for scoliotic curves with CA > 40° was found to be stronger than for smaller curvatures [28].

##### Other techniques combined

When all correlation effect sizes from the category of “other techniques” were combined, the correlation with radiographic CA was moderate ($r=0.77, 95 \% CI:[0.67, 0.84]$) (Supplementary Table 9).

#### Validity overview of radiation-free scoliosis assessment categories

**Fig. 8:**
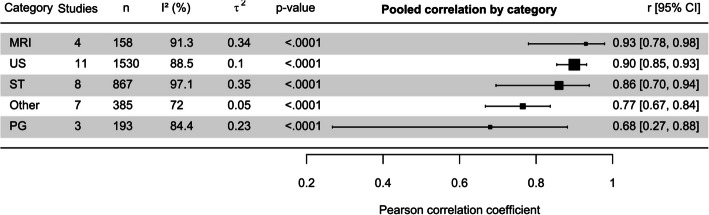
Forest plot displaying pooled overall Pearson correlation coefficient by category (magnetic resonance imaging (MRI), ultrasonography (US), surface topography (ST), photogrammetry (PG)), along with the number of included studies and scoliosis patients per pooled category. τ^2^ values represent the between-study variance components in the multilevel meta-analysis models, I^2^ quantifies the proportion of variability in effect estimates due to heterogeneity rather than sampling error, and the *p*-value shows the level of significance of the heterogeneity test

Overall, MRI showed the strongest pooled correlation with radiographic examination in terms of coronal CA, followed by strong correlations of US and ST, and moderate correlation in the photogrammetry and “other techniques” categories (Fig. 8).

The I2 values ranged from 72% to 97.1% across categories, indicating substantial heterogeneity in all categories. ST showed the highest heterogeneity (I2 = 97.1%), suggesting that 97.1% of the variability in effect sizes was due to differences between studies rather than sampling error. However, the lowest I2 value also signifies considerable heterogeneity. τ2 values indicate the extent of variance between studies, where higher τ2 values in ST (0.35) and MRI (0.34) reflected greater inter-study variance in these categories. Lower τ2 e.g. in the US category suggested relatively less variance in effect sizes.

## Discussion

This systematic review of the literature offers a comparative overview of techniques that could potentially reduce the frequency of X-ray examinations for scoliosis monitoring. In terms of validity, most radiation-free assessment modalities demonstrated moderate to strong correlations with radiographically derived Cobb angles, the current clinical gold standard. Three techniques (MRI, ultrasonography, and surface topography) exhibited strong to very strong correlations (*r* > 0.8) with this gold standard. Among these, ultrasonography and surface topography were the most extensively studied in terms of publications and patient data. Despite potentially high validity levels, inconsistent results were reported in the MRI category. Two-dimensional photogrammetry and methods grouped under the “other techniques” category (including scoliosis assessment using spatial landmark mapping, Spinal Mouse, motion analysis, or inclinometers) showed only moderate agreement with radiographic assessment and were less frequently investigated. Based on these findings, ultrasonography and surface topography stand out as the most promising techniques for radiation-free scoliosis monitoring from an overall and validity perspective, offering considerable potential to minimize patients’ exposure to ionizing radiation. Moreover, this systematic review of the literature highlights the ongoing need for longitudinal follow-up studies to validate radiation-free technologies in monitoring scoliosis progression and treatment efficacies.

Ultrasound demonstrated the most consistent correlations with standing radiographs in the coronal plane, which was reasonable since this modality allows actual imaging of dorsal anatomical spine structures with patients in an upright standing posture—similar to radiographic examinations. Other modalities were either limited to Superficial imaging or required patients to be in Supine postures, e.g. lying during MRI. The mean absolute difference of 3.4° was below the commonly used radiographic CA threshold of 5° for the detection of deformity progression [9, 22, 27, 33, 61], hence fulfilling the precondition for use in clinical monitoring. Of the different dorsal vertebral structures from which the curvature angle could be derived within ultrasound imaging, use of the transverse processes correlated best with the radiographic CA. The transverse processes (as the vertebral structures most distant from one-another) seem to provide the most reliable basis for accurate CA measurements). However, the accurate localization of transverse processes remains difficult due to the generally indistinct appearance of bony structures in ultrasound images. Conversely, spinous processes are commonly identifiable vertebral structures, but axial rotation can hardly be captured using these landmarks, hence rendering their use for reliable estimates of CAs difficult [39]. A consistent agreement between ultrasound measurements and corresponding radiographic measurements in the sagittal plane, as well as for detecting deformity progression using ultrasound, remains to be demonstrated, with each supported by only two studies.

While the information obtained from US assessments is limited to the immediate vicinity of the spine, whereas the strength of surface topography is the wide field of view possible. Thus, scoliosis-induced chest wall deformities like a prominent scapula, unleveled shoulders, rib hump asymmetry, as well as pelvic obliquity and waistline deformities [91, 92] can all be factored into the evaluation to estimate spinal parameters, including Cobb angle. Using this technique, curvatures were more accurately assessed in the thoracic compared to lumbar regions. ST approaches using non-certified generic or self-built scanning setups performed slightly better than the rasterstereographic measurements by the certified DIERS formetric 4D system [93]. This is possibly due to regulations around certification limiting small iterative improvements to the technology. Our review of the literature found a consistent correlation for both the thoracic kyphosis angle and lumbar lordosis angle in the sagittal plane (r≈0.8), which was higher than reported previously [93] (TKA: *r* = 0.75, 95% CI [0.64, 0.83]; LLA: *r* = 0.71, 95% CI [0.57, 0.82]). These results suggest that this technology continues to evolve, with particularly promising developments in data-driven evaluation of surface topography [59].

MRI-derived CAs showed a very strong overall correlation with their radiographic counterparts. However, patient positioning played a considerable role on the correlation with standing radiography. In a Supine posture, Cobb angles were reported to be on average 5° to 7.7° lower than radiographic CAs [80, 81, 94], and the use of an axial loading device to simulate gravitational forces during upright standing was proposed [81]. Upright positioning MRI, on the other hand, where gravitational loading was consistent with standing radiography, has also demonstrated consistent and strong correlations. This inconsistency in imaging posture has likely introduced the large observed heterogeneity between study results (cf. Fig. 8). Despite its superior imaging quality, the time requirements, limited availability, and high costs associated with full spine MRI examinations seem to be considerable drawbacks [80, 95]. These factors, alongside the different spinal postures in prone or Supine positions compared to standing, Likely contribute to why MRI has not become established as a standard method for monitoring scoliosis. However, recent advancements in real-time full-spine MRI protocols with examination times of less than 10 min on average might place MRI as a more viable alternative to radiography for scoliosis monitoring in the future [45].

Photogrammetry demonstrated highly variable correlation coefficients, ranging from 0.4 to over 0.9, with a MAD of 4.1° [74, 77]. This method is Limited to assessments in the coronal plane due to its 2D nature. The accuracy of photogrammetry often critically depends on the rater’s expertise, as the technique requires precise detection or palpation of anatomical landmarks and careful placement of custom markers. Although photogrammetry benefits from low technology requirements, its data output is less comprehensive, as it lacks topographic depth information. Consequently, this limitation restricts the data richness and narrows the scope of evaluation possibilities, potentially affecting its validity when compared to methods that provide more detailed three-dimensional information.

The approaches summarized in the category of “other techniques” spatially map palpated anatomical landmarks of the back surface —primarily the spinous processes—to estimate spinal parameters. These mappings are performed in habitual upright standing and/or various bending postures, such as forward flexion (Scoliometer) and lateral flexion (motion analysis). The correlation with radiographically determined Cobb angle reached moderate up to strong values in certain cases (*r* = 0.69 to 0.93), with MADs between 4° to 7°. However, a significant limitation of these approaches is their limited data content, as only specific points of the back surface in upright standing or bending positions are recorded. Vertebral rotation around the craniocaudal axis may not be adequately captured [83], thus often underestimating the true Cobb angle. Importantly, the data obtained from these methods are a Subset of a more comprehensive data collection setup using e.g. ST, which uses 3D scanning to provide trunk or back surface reconstructions [96–98]. For instance, in the case of spatial landmark mapping, points on the back surface are palpated and their spatial positions measured; the Spinal Mouse records the line connecting each spinous process; inclinometers measure angles relative to gravity; and motion analysis maps markers in space. Given the restricted data content and limited scope of measurement, it is plausible that the validity and accuracy achievable by these “other techniques” is inferior to that of ST setups allowing for more comprehensive data collection, as corroborated by the data collected within this systematic review (cf. Fig. 8).

Novel emerging data-driven approaches hold promise to considerably advance the validity of radiation-free scoliosis monitoring. For instance, the use of 2D back images alone to estimate scoliotic curvature progression has demonstrated a predictive accuracy of 70% [78]. Moreover, synthesizing X-ray images from US or ST data for direct comparison of CAs against those from clinical radiographs has also shown very strong correlations, as demonstrated in a large ST study (*R*^2^ = 0.984, *n* = 302) [59], as well as in a study using US (*r* = 0.95, *n* = 42) [67]. The key advantage of synthesizing X-ray images from radiation-free methods is that the determinations remain consistent with those used in clinical X-ray examinations, ensuring direct comparability. Interestingly, only one of the included studies [99] utilized patient-specific insights from initial diagnostic radiographs to enhance the accuracy of subsequent radiation-free monitoring assessments. This progressive approach to integrate data from previous examinations seems prudent, especially since replacing scoliosis diagnosis with a radiation-free technique is not currently allowable. However, considering the high agreement between radiographic examination and certain radiation free methods, especially US, ST, and MRI, integration of these techniques into clinical monitoring with the aim of reducing patients’ exposure to ionizing radiation seems to be increasingly feasible. As a result, radiographic examinations could be prescribed only if e.g. radiation-free monitoring indicates a progression of the spinal deformity. Alternatively, the frequency of radiographic monitoring could be reduced, while radiation-free monitoring is applied for interim evaluations. Thus, changes in deformity could be detected earlier without the detrimental effects of ionizing radiation. However, to this end, it is crucial to consider aspects beyond diagnostic accuracy alone; while their broader consideration was beyond the scope of this review, aspects such as cost-effectiveness, time requirements, device availability, regulatory certification (e.g., FDA approval or CE marking), ease of use, reliability, necessary training, and operational challenges all need to be overcome before radiation-free monitoring can be integrated into clinical settings. Furthermore, each radiation-free modality comes with its own limitations regarding patient selection and outcomes, aspects that have also not been discussed in detail in this review. In addition, it is important to note that during treatment, radiographs are often performed in-brace to evaluate brace fit and treatment response. Most radiation-free imaging modalities reviewed here are not suitable or validated for in-brace assessment, limiting their applicability and potential to reduce radiation exposure in this context, where plain radiographs remain essential.

Further consideration must also be given to modernization and technical advances in radiographic imaging that have significantly contributed to reducing radiation exposure. Reported effective radiation doses of conventional full-spine digital radiography vary widely, ranging from 0.07 to 2.7 millisieverts (mSv) across different studies [16, 100–104]. This variability can be attributed to numerous factors, including differences in manufacturers and imaging units, year of manufacture, imaging protocols and settings, and patient-specific characteristics Such as body habitus. In contrast, slot-scanning radiographic systems Such as the EOS system have demonstrated lower radiation doses. The effective dose for a standard-dose full-spine EOS examination has been reported to range between 0.09 and 0.22 mSv [100, 105], while the micro-dose mode reduces this further to 0.0025 to 0.017 mSv [101, 105, 106]. These Substantial dose reductions translate into a significantly lower iatrogenic cancer risk, particularly with micro-dose EOS, which achieves a 16- to 40-fold decrease in effective dose and was therefore recommended for repeated patient follow-up [101, 105]. Dual-energy X-ray absorptiometry (DXA), primarily used to assess bone mineral density but occasionally applied in the evaluation of spinal deformity [107], also involves very low radiation doses, comparable to those of micro-dose EOS imaging [108]. For perspective, the average annual effective dose from natural background radiation is estimated to be approximately 3.0 mSv [109]. Nonetheless, the relevance of radiation-free monitoring methods extends beyond dose reduction. Access to advanced low-dose systems Like EOS is not universal, particularly in resource-limited settings. Radiation-free techniques may also enable decentralized or more frequent monitoring in the future. For example, one could envision a hypothetical scenario in which smartphone-based 3D scanning is used at home to track spinal changes and trigger radiographic evaluation only if progression is suspected, thereby reducing clinical burden and cumulative radiation exposure while maintaining effective follow-up.

There was no apparent risk of bias for the index test (radiation-free modality) and reference standard recorded, i.e. results of the index test were interpreted by raters without knowledge of the results of the reference standard and vice versa, and assessor experience was sufficient. However, the selection of patients exhibited considerable bias across the reviewed studies. Common exclusion criteria included a body mass index (BMI) or Cobb angles exceeding predefined threshold values, certain types of curvatures, and modality-specific exclusions (Supplementary Table 1). Significant gender disparities not justifiable by prevalence differences between sexes were also often observed. These exclusion criteria Suggest that assessment results obtained from radiation-free methods may not be reliable for AIS patients with a BMI over 24 or a Cobb angle greater than 40°, although the degree to which BMI affects measurement accuracy likely varies across imaging modalities and is not consistently reported. Given the lack of robust evidence supporting the validity of radiation-free scoliosis monitoring methods for such patients, reliance on such methods for follow-up is not recommended. Furthermore, we strongly recommend reporting demographic and/or anthropometric data, as well as the time interval between the index test and the reference standard, as these details are often omitted in reporting. Assessment delays could allow for curve progression, which may act as a confounding factor and limit the validity of the reported results [110].

The focus on monitoring rather than direct diagnosis generally stems from the recognition of health concerns associated with repeated X-ray exposure over time. While initial diagnosis typically occurs just once and must be precise and allow for the evaluation of the exact morphology of the deformity during standing, monitoring requires recurring evaluations, often at intervals ranging from six months to a year [12]. However, many of the included studies did not explicitly deploy a follow-up protocol for scoliotic patients. Instead, they introduced or utilized radiation-free modalities and investigated validity and/or accuracy compared to the reference standard in a cross-sectional setup. Often the modalities were proposed for use in monitoring scenarios with the aim of reducing radiographic examinations, but not actually tested in Such a setup. Among the included studies, only 15 out of 56 applied a radiation-free scoliosis assessment technique in a longitudinal monitoring setup, reflecting the applicability concerns highlighted in the quality assessment. This underscores a significant gap in the field: the lack of longitudinal studies involving radiation-free scoliosis monitoring, an issue that future research should address to enhance patient care and reduce reliance on invasive monitoring procedures. Out of the 15 studies that incorporated follow-up procedures in their clinical investigations, only five reported that using a radiation-free modality could potentially reduce the number of radiographs required for scoliosis monitoring, with the proposed reduction ranging from 30 to 71% [27, 33].

The substantial heterogeneity observed across all radiation-free categories suggests that the studies included in each category differed in terms of methodologies, patient populations, measurement tools, or other factors, indicating a lack of consensus. It is therefore important to note that such heterogeneity might impact the generalizability of the results and could potentially affect the statistical power of the meta-analysis. Validity and accuracy estimates pooled from only a small number of studies should therefore be interpreted with caution, as these might be biased. Additionally, although the search strategy was developed iteratively and aimed for comprehensive coverage, the use of exclusion terms (e.g., via the ‘NOT’ operator) to increase specificity may have inadvertently led to the omission of relevant studies. A review protocol was prepared for this systematic review; however, the review was not registered and the protocol not published.

## Conclusions

This systematic review of the Literature analyzed 56 studies focusing on non-invasive, radiation-free devices and techniques that were used or proposed for monitoring scoliosis, with comparisons against radiographic examination.

Ultrasonography emerged as the most robust method, demonstrating a very strong pooled correlation with radiographic imaging (r≈0.9) and offering accurate Cobb angle estimates with minimal deviations. Surface topography also showed a strong pooled correlation (*r* > 0.8) with radiographic examinations. Both technologies could significantly reduce the reliance on radiographic examinations in scoliosis monitoring for a restricted scoliosis patient population. Their application may be Limited in patients with a BMI over 24 or a Cobb angle greater than 40°, where the validity of radiation-free assessments have yet to be adequately demonstrated.

For eligible patients, integrating ultrasonography or surface topography into routine scoliosis monitoring could become feasible in the future. Potential applications include using these radiation-free technologies to monitor changes in spinal curvature, with radiographic examinations reserved for confirming significant deformity progression. Alternatively, these technologies could be used more frequently (e.g., every 3 to 6 months), while radiographic examinations could be spaced further apart, thus minimizing exposure to ionizing radiation.

While many studies promote the use of radiation-free methods for scoliosis monitoring, few have demonstrated their effectiveness in longitudinal follow-up studies. Longitudinal follow-up studies are necessary to create sufficiently strong evidence, and the establishment of robust protocols is needed to truly shift the paradigm in scoliosis monitoring.

## Supplementary Information

Supplementary Material 1.

## Data Availability

All data supporting the conclusions of this article were extracted from publicly available scientific publications, which are cited in the reference list. A summary of the data can be found in the supplementary material.

## Abbreviations

AIS: Adolescent idiopathic scoliosis
CA: Cobb angle
CI: Confidence interval
MAD: Mean absolute difference
mSv: Millisievert
MRI: Magnetic resonance imaging
PG: Photogrammetry
SD: Standard deviation
Se: Sensitivity
Sp: Specificity
ST: Surface topography
US: Ultrasonography
