# Supplementary material for: Exploring radiation-free scoliosis monitoring: systematic review and meta-analysis of non-ionizing methods
Source: BMC Musculoskelet Disord. 2025 Oct 1;26:899. doi: 10.1186/s12891-025-09034-8 (PMC12487184; doi:10.1186/s12891-025-09034-8)
Supplement: Supplementary file 1 — Supplementary Material 1. [file 12891_2025_9034_MOESM1_ESM.docx]

Supplementary materials

For the publication titled “Exploring radiation-free scoliosis monitoring: systematic review and meta-analysis of non-ionizing methods” by Bertsch M., et al.

## Example search query (Pubmed)

(scolio*[tiab])

AND

("non invasive*"[tiab] OR "non ionizing"[tiab] OR "non-invasive*"[tiab] OR "non-ionizing"[tiab] OR "radiation free"[tiab] OR "radiation-free"[tiab] OR "non-radiographic"[tiab] OR "ionization free"[tiab] OR noninvasive*[tiab] OR nonionizing[tiab] OR radiation[tiab] OR "x-ray"[tiab] OR ionizing[tiab] OR radiograph*[tiab] OR cobb[tiab])

AND

(analys*[tiab] OR assess*[tiab] OR check*[tiab] OR evaluat*[tiab] OR examin*[tiab] OR follow*[tiab] OR measur*[tiab] OR monitor*[tiab] OR observ*[tiab] OR record*[tiab] OR stud*[tiab] OR surveil*[tiab] OR track*[tiab] OR diagnos*[tiab] OR screen*[tiab])

AND

(scan*[tiab] OR MRI[tiab] OR alternative[tiab] OR imaging[tiab] OR intelligen*[tiab] OR learning[tiab] OR network*[tiab] OR optical[tiab] OR photogrammetry[tiab] OR rasterstereography[tiab] OR resonance[tiab] OR stereoscop*[tiab] OR substitut*[tiab] OR surrogate[tiab] OR thermograph*[tiab] OR topograph*[tiab] OR ultrasonography[tiab] OR ultrasound[tiab] OR inclinometer[tiab] OR scoliometer[tiab] OR motion[tiab] OR "spinal mouse"[tiab])

NOT

(surgery[tiab] OR tethering[tiab] OR "spinal fusion"[tiab] OR animal[tiab] OR mice[tiab] OR cadaver*[tiab] OR pregnan*[tiab])

AND

("2000/01/01"[PDAT] : "2024/08/31"[PDAT])

## Categorization of radiation-free scoliosis monitoring techniques

This review categorizes five primary radiation-free techniques used for scoliosis monitoring based on their technological basis:

**Supplementary Table 1**: Summarizes the various non-invasive, radiation-free devices and methods used for monitoring scoliosis, detailing their methodologies and key devices/systems.

| **Technology** | **Description/Methodology** | **Key devices/systems** |
| --- | --- | --- |
| Surface topography (ST) | Digitizes the 3D shape of the thorax or back, e.g., by projecting parallel white light stripes, capturing them with a camera, and using triangulation to determine spatial coordinates. This process is also termed rasterstereography. Spinal and/or spinopelvic parameters, for example the Cobb angle or vertebra rotation, are estimated through back shape analysis. | DIERS Formetric 4D  DIERS Statico 3D  BIOMOD-L  Generic 3D scanners  Self-developed systems |
| Ultrasonography (US) | Employs ultrasound imaging to visualize posterior vertebral structures, from which spinal parameters to quantify scoliosis can be obtained. Often volume projection imaging is used to create a 3D volume from transverse ultrasound imaging slices. | SCOLIOSCAN  Generic ultrasound probes |
| Magnetic resonance imaging (MRI) | Visualizing internal soft and hard tissue structures for scoliosis assessment, mostly in supine positioning with or without axial loading to simulate the gravitational load situation of the spinal column during upright standing. | MRI devices, upright positional MRI |
| Photogrammetry (PG) | Concentrates on 2D-image-based measurements to obtain spinal parameters and quantify deformities, using commercial 2D cameras often in conjunction with surface markers and palpation procedures. Involves no 3D reconstruction (contrary to photogrammetry applications in the context of computer vision). | Generic 2D cameras |
| Other | In this category methods were summarized that are less used for scoliosis monitoring. These comprise inclinometers (e.g. the scoliometer) to measure deformity-related back surface angles against gravity, spatial landmark mapping that maps the spatial coordinates and relation of often manually palpated anatomical landmarks on the back surface (mainly the palpated locations of the spinous processes), the Spinal Mouse that utilizes the line along the spinous processes on the back surface, and motion capture systems to also map locations of anatomical landmarks in space for deformity quantification. | Scoliometer/Inclinometer Spinal Mouse  Motion capture systems  ZEBRIS Spine  Ortelius 800 |

## Study characteristics and population demographics


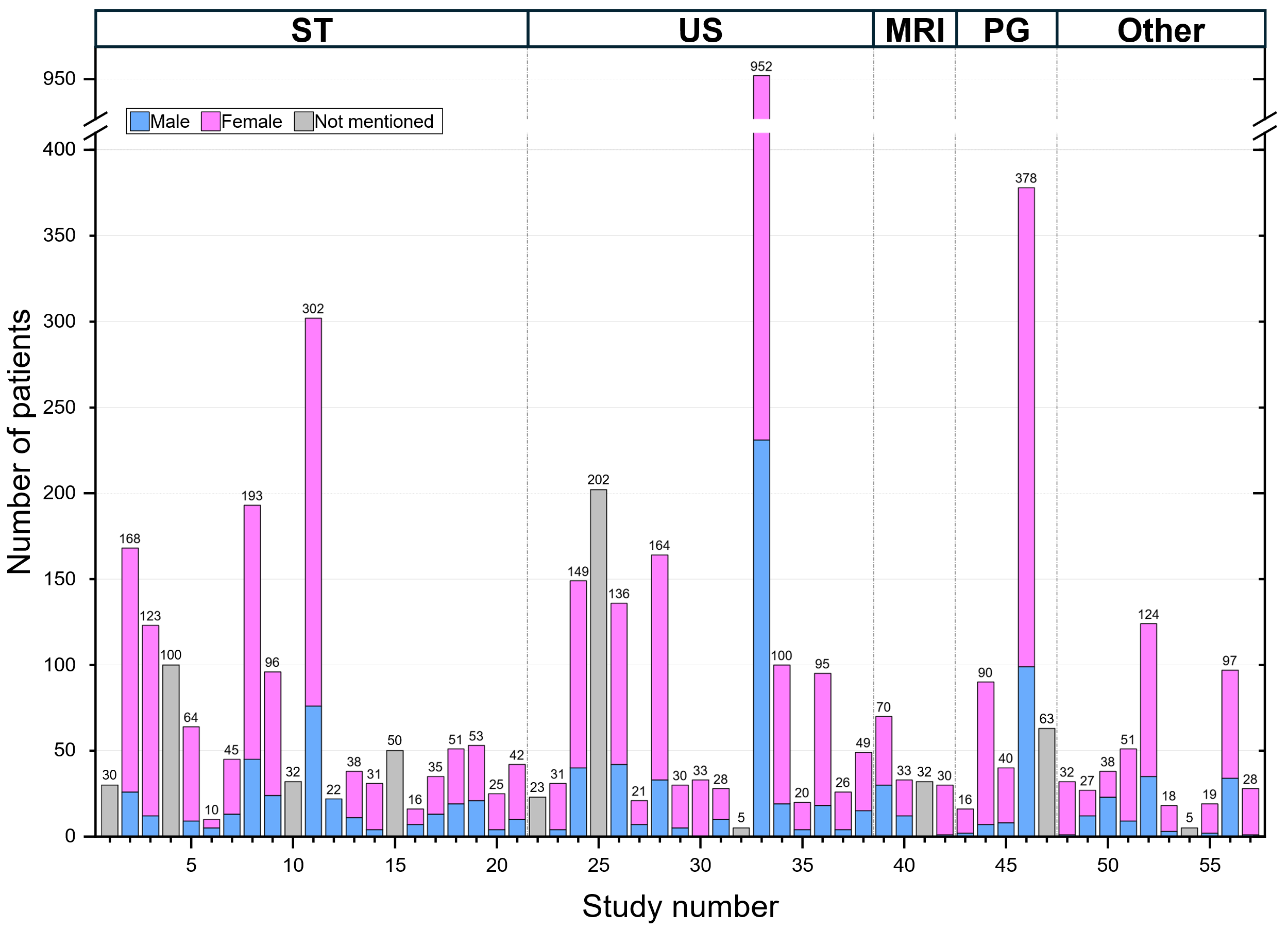


**Supplementary Figure 2**: Number of patients per study by category, along with the gender ratio. Study number as per Supplementary Table 1, for full reference see main bibliography.

**Supplementary Table 2**: Characteristics of the participants of studies included in the systematic review divided by techniques (*Values with an asterisk have been calculated by the reviewers; SC = scoliosis group, HC = healthy controls, NA = not available; M = males; F = females; CA = radiographic Cobb angle; BMI = body mass index [kg/m^2^], (A)IS = (adolescent) idiopathic scoliosis, LLD = leg length discrepancy [cm]). Reference numbers see bibliography of publication.

|  | **NO.** | **STUDY [Ref.]** | **SC** | **HC** | **F** | **M** | | | **MEAN AGE (SC)** | **INCLUSION** | **EXCLUSION** |
| --- | --- | --- | --- | --- | --- | --- | --- | --- | --- | --- | --- |
| ST | 1 | Adankon (2013) [22] | 30 | 28 | NA | | | | NA | AIS, age 10-18, CA>10° | Brace treatment |
|  | 2 | Berryman (2008) [56] | 168 |  | 142 | 26 | | | 15.25 | NA | NA |
|  | 3 | Bolzinger (2021) [27] | 123 |  | 111 | 12 | | | 12 | AIS, age 10-13, CA>10° | CA>40°, BMI>25 |
|  | 4 | De Korvin (2014) [31] | 100 |  | NA | | | | 13.3 ± 2.1 | CA>10° |  |
|  | 5 | Frerich (2012) [57] | 64 |  | 55 | 9 | | | 13* | AIS | CA>50, BMI>20 |
|  | 6 | Grünwald (2022) [98] | 10 |  | 5 | 5 | | | 21 | AIS, neurogenic scol. |  |
|  | 7 | Hong (2017) [61] | 45 |  | 32 | 13 | | | 13.8 | AIS, age 8-18, CA>10° | CA>45° |
|  | 8 | Knott (2016) [44] | 193 |  | 148 | 45 | | | 13.25 | AIS, age 8-18, CA≥10° | CA>50°, |
|  | 9 | Komeili (2015) [9] | 96 |  | 72 | 24 | | | NA | BMI>25, double curves |  |
|  | 10 | Liang (2024) [54] | 32 |  | NA | | | | NA | NA | NA |
|  | 11 | Meng (2023) [59] | 302 |  | 226 | 76 | | | 14* | AIS, age 10-18 | dermatological cond. |
|  | 12 | Mishra (2020) [60] | 22 |  | 0 | 22 | | | 21.5* | NA | NA |
|  | 13 | Patel (2024) [62] | 38 |  | 27 | 11 | | | 13.8 | IS, age 8-18 |  |
|  | 14 | Pino-Almero (2016) [33] | 31 |  | 27 | 4 | | | 13 | AIS, CA>10° |  |
|  | 15 | Rothstock (2023) [55] | 50 |  | NA | | | | 13.5* | IS, age 12-15 |  |
|  | 16 | Schulte (2008) [34] | 16 |  | 9 | 7 | | | 13 | ≥3 examinations in 3 yrs | X-ray w/o brace |
|  | 17 | Tabard‑Fougère (2017) [42] | 35 |  | 22 | 13 | | | 13.1 ± 2.0 | AIS, age 10-18, CA>10° | BMI≥29, tattoos, CA<40° |
|  | 18 | Tabard‑Fougère (2019) [29] | 51 |  | 32 | 19 | | | 13.5 ± 2.0 | AIS, age 10-18, CA>10° | BMI≥29, tattoos |
|  | 19 | Tabard‑Fougère (2023) [43] | 53 |  | 32 | 21 | | | 13.5 ± 1.9 | AIS, age 10-18, CA>10° | BMI≥29, tattoos |
|  | 20 | Weiss (2013) [58] | 25 |  | 21 | 4 | | | 12.9 | AIS | Brace treat. |
|  | 21 | Yildirim (2021) [90] | 42 |  | 32 | 10 | | | 14.1 | AIS | Certain anatom. profiles |
| US | 22 | Chen (2024) [69] | 23 |  | NA | | | | 13.3 ± 2.6 | NA | NA |
|  | 23 | Ferràs‑Tarragó (2019) [65] | 31 |  | 27 | | | 4 | NA | AIS, age 10-18 | CA>45° |
|  | 24 | Huang (2024) [99] | 149 |  | 109* | | | 40* | 21.6 | Testing set: age 10-14 |  |
|  | 25 | Jiang (2022) [67] | 202 |  | NA | | | | 16.2 |  | Metallic implants, brace treatment, BMI>25 |
|  | 26 | Lai (2023) [71] | 136 |  | 94 | 42 | | | 14.1 ± 1.9 | AIS, age 10-18 | Metallic implants, braces, BMI>30 |
|  | 27 | Lee TT. (2019) [73] | 21 |  | 14 | 7 | | | 15.7 ± 1.3 | AIS | Metallic implants, BMI>25, CA>50° |
|  | 28 | Lee TT. (2021) [41] | 164 |  | 131 | 33 | | | 15.1 ± 1.9 | AIS, age 10-18 | Met. implants, BMI>25 |
|  | 29 | Li DS. (2020) [72] | 30 |  | 25 | 5 | | | 13.4 ± 5.6 | NA | NA |
|  | 30 | Li M. (2015) [64] | 33 |  | 33 | 0 | | | NA | AIS, female, CA>10°, age 9-14, Risser sign ≤ 2 | CA>40° |
|  | 31 | Lv (2020) [66] | 28 | 8 | 18 | 10 | | | 17.7 ± 1.4 | AIS | CA>45°, BMI>25, metallic implants |
|  | 32 | Wong (2019) [39] | 952 |  | 721 | 231 | | | 16.7 | IS, age 8-40, BMI<23 | winged scapula, implants |
|  | 33 | Yang (2022) [40] | 100 |  | 81 | 19 | | | 15 ± 1.9 | AIS |  |
|  | 34 | Young (2015) [70] | 20 |  | 16 | 4 | | | 14.5 ± 1.7 | AIS, CA>10° | CA>45° |
|  | 35 | Zeng (2024) [68] | 95 |  | 77 | 18 | | | 14*±*2 | CA>10° | CA>50° |
|  | 36 | Zheng R. (2015) [63] | 26 |  | 22 | 4 | | | 13.9 ± 2.1 | AIS, CA>10° | CA>45° |
|  | 37 | Zheng YP. (2016) [37] | 49 |  | 34 | 15 | | | 15.8 | AIS, BMI<25 | winged scapula, implants |
| MRI | 38 | Diefenbach (2013) [79] | 25 |  | 16 | 9 | | | 14.6 | AIS |  |
|  | 39 | Lee MC. (2013) [80] | 70 |  | 40 | | 30 | | 14 | AIS, age 10-18 | LLD>2 |
|  | 40 | Roth (2024) [45] | 33 |  | 21 | | 12 | | NA | IS, age 5-17 |  |
|  | 41 | Schmitz (2001) [94] | 32 | 18 | NA | | | | 14.8 | IS |  |
|  | 42 | Wessberg (2006) [81] | 30 |  | 29 | 1 | | | 13.6 | AIS, age 12-16, brace |  |
| PG | 43 | Aroeira (2011) [77] | 16 |  | 14 | 2 | | | 21.4 ± 6.1 | IS |  |
|  | 44 | Leal (2019) [74] | 90 |  | 83 | 7 | | | 14.03 | AIS |  |
|  | 45 | Saad (2009) [76] | 40 |  | 32 | 8 | | | 23.4 ± 11.2 | IS | CA<10°, LLD |
|  | 46 | Zhang (2023) [78] | 378 |  | 279 | | 99 | | 14.3 ± 3.8 | AIS, age 10-18 | BMI>30 |
|  | 47 | Zheng Q. (2023) [75] | 63 | 47 | NA | | | | 13 ± 1 | AIS, CA>10°, age 10-16 | CA>45°, LLD |
| Other | 48 | Coelho (2013) [89] | 32 |  | 31 | 1 | | | 18.2 ± 3.9 | IS, age 10-25 | LLD>2.5 |
|  | 49 | Kim (2024) [87] | 38 |  | 15 | 23 | | | 12.9 *±* 2.5 | AIS, CA>10° | Brace treatment |
|  | 50 | Livanelioglu (2016) [28] | 51 |  | 42 | 9 | | | 14.4 | AIS, age 9-18 | skin folds or scars |
|  | 51 | Ovadia (2007) [82] | 124 |  | 89 | 35 | | | 13 | AIS | CA>55° |
|  | 52 | Salvia (2022) [85] | 18 | 15 | 15 | | 3 | | 13.8 ± 1.5 | IS, CA | LLD>1.2 |
|  | 53 | Solomito (2011) [86] | 5 | 5 | NA | | | | NA | AIS |  |
|  | 54 | Takacs (2018) [84] | 19 |  | 17 | 2 | | | 14.2 | AIS, age 8-16 |  |
|  | 55 | Wei (2023) [88] | 97 |  | 63 | 34 | | | 14 | Age 10-17 | LLD |
|  | 56 | Zabka (2015) [83] | 28 |  | 27 | 1 | | | NA | NA | NA |

## Quality assessment

**Supplementary Table 3**: Criteria that were applied when assessing the quality of included studies in the four domains.

| **Domain** | **Risk of bias was identified in case of:** | **Concerns of applicability were indicated in case of:** |
| --- | --- | --- |
| **Patient selection** | - indication of non-consecutive or non-random patient enrolment - exclusions based on age, gender, BMI, Risser sign, the degree of the curvature, or modality-specific exclusions - strongly imbalanced genders, unclear patient selection, large age differences, low participant numbers (n<10) or missing power analysis | - ambiguous or unspecific diagnosis, for example only ”scoliosis” without further specification - a lack of demographic data on subjects (the term “AIS” was considered as age demographic data) |
| **Index test** | - interpretation of results with knowledge of reference standard results (X-ray) - insufficient operator/assessor experience (e.g., medical students) - lack of pre-defined thresholds (if there are thresholds in the method) | - no follow-up procedure (follow-up = at least two monitoring intervals) |
| **Reference standard** | - insufficient operator/assessor experience - interpretation of results with knowledge of index test results | - unclear definition of the radiograph assessment - lack of deformity progression thresholds |
| **Flow and timing** | - time between index test and reference standard examination more than 1 month apart or no information given. - monitoring intervals below 3 months or beyond 2 years - not all recruited patients included in analysis |  |

**Supplementary Table 4**: Detailed results of the quality assessment of included studies by category as per the QUADAS-2 tool. Each study was evaluated in terms of concerns of applicability and risk of bias, and rated with a high (☹), moderate (😐), low (☺), or unclear (.?.) bias risk or applicability concern, respectively. Study references: see Table 2.

| **CAT.** | **STUDY** | **CONCERN OF APPLICABILITY** | | | **RISK OF BIAS** | | | |
| --- | --- | --- | --- | --- | --- | --- | --- | --- |
|  |  | **Patient selection** | **Index test** | **Reference standard** | **Patient selection** | **Index test** | **Reference standard** | **Flow & Timing** |
| ST | Adankon (2013) | ☺ | ☺ | ☺ | ☹ | ☺ | ☺ | 😐 |
|  | Berryman (2008) | ☹ | ☺ | ☺ | 😐 | ☺ | ☺ | .?. |
|  | Bolzinger (2021) | ☺ | ☺ | ☺ | ☹ | .?. | ☺ | 😐 |
|  | De Korvin (2014) | ☹ | .?. | ☺ | ☺ | ☺ | ☺ | 😐 |
|  | Frerich (2012) | ☺ | ☹ | ☺ | ☺ | ☺ | ☺ | ☺ |
|  | Grunwald (2022) | ☹ | ☹ | ☺ | ☺ | ☺ | ☺ | 😐 |
|  | Hong (2017) | ☺ | ☺ | ☺ | 😐 | ☺ | ☺ | 😐 |
|  | Knott (2016) | ☺ | ☹ | ☺ | 😐 | ☺ | ☺ | ☺ |
|  | Komeili (2015) | ☺ | ☺ | ☺ | 😐 | ☺ | ☺ | ☹ |
|  | Liang (2024) | ☹ | ☹ | ☺ | .?. | .?. | .?. | 😐 |
|  | Meng (2013) | ☺ | ☹ | ☺ | 😐 | ☺ | ☺ | ☺ |
|  | Mishra (2020) | ☺ | ☺ | ☺ | 😐 | ☺ | ☺ | 😐 |
|  | Patel (2024) | ☺ | ☺ | ☺ | ☺ | ☺ | ☺ | ☹ |
|  | Pino-Almero (2016) | ☺ | ☺ | ☺ | ☺ | ☺ | ☺ | 😐 |
|  | Rothstock (2023) | ☺ | ☹ | ☺ | 😐 | ☺ | ☺ | ☺ |
|  | Schulte (2008) | ☺ | ☺ | ☺ | ☺ | ☺ | ☺ | ☺ |
|  | Tabard‑Fougère (2017) | ☺ | ☹ | ☺ | 😐 | ☺ | ☺ | 😐 |
|  | Tabard‑Fougère (2019) | ☺ | ☹ | ☺ | 😐 | ☺ | ☺ | ☺ |
|  | Tabard‑Fougère (2023) | ☺ | ☹ | ☺ | 😐 | ☺ | ☺ | ☺ |
|  | Weiss (2013) | ☺ | ☹ | ☺ | ☺ | ☺ | ☺ | ☺ |
|  | Yildirim (2021) | ☺ | ☺ | ☺ | ☺ | ☺ | ☺ | ☺ |
| US | Chen (2024) | ☹ | ☹ | ☺ | .?. | ☺ | ☺ | 😐 |
|  | Ferras-Tarrago (2019) | ☺ | ☹ | ☺ | 😐 | ☺ | ☺ | 😐 |
|  | Huang (2024) | ☺ | .?. | .?. | 😐 | ☺ | 😐 | ☹ |
|  | Jiang (2022) | ☺ | ☹ | ☺ | 😐 | .?. | 😐 | ☺ |
|  | Lai (2023) | ☺ | ☺ | ☺ | ☺ | ☺ | ☺ | ☺ |
|  | Lee TT. (2019) | ☺ | ☹ | ☺ | 😐 | ☺ | ☺ | 😐 |
|  | Lee TT. (2021) | ☺ | ☹ | ☺ | 😐 | ☺ | ☺ | ☺ |
|  | Li DS. (2020) | ☹ | ☹ | ☺ | 😐 | ☺ | ☺ | 😐 |
|  | Li M. (2015) | ☹ | ☹ | ☺ | 😐 | ☺ | ☺ | 😐 |
|  | Lv (2020) | ☺ | ☹ | ☹ | 😐 | ☺ | 😐 | 😐 |
|  | Wong (2019) | ☺ | ☹ | ☺ | 😐 | ☺ | ☺ | 😐 |
|  | Yang (2022) | ☺ | ☹ | ☺ | ☺ | ☺ | ☺ | ☺ |
|  | Young (2015) | ☺ | ☺ | ☺ | 😐 | ☺ | ☺ | ☺ |
|  | Zeng (2024) | ☺ | ☹ | ☺ | ☺ | ☺ | ☺ | .?. |
|  | Zheng R. (2015) | ☺ | ☹ | ☺ | 😐 | ☺ | ☺ | 😐 |
|  | Zheng YP. (2016) | ☹ | ☹ | ☺ | 😐 | ☺ | ☺ | ☺ |
| MRI | Diefenbach (2013) | ☺ | ☹ | ☺ | ☺ | ☺ | ☺ | ☺ |
|  | Lee MC. (2013) | ☺ | ☺ | ☺ | ☺ | ☺ | ☺ | ☺ |
|  | Roth (2024) | ☺ | ☹ | ☺ | ☺ | ☺ | ☺ | 😐 |
|  | Schmitz (2001) | ☺ | ☹ | ☺ | ☺ | ☺ | ☺ | 😐 |
|  | Wessberg (2016) | ☺ | ☺ | ☺ | 😐 | ☺ | ☺ | ☺ |
| PG | Aroeira (2011) | ☺ | ☹ | ☺ | ☺ | ☺ | ☺ | ☺ |
|  | Leal (2019) | ☺ | ☺ | ☺ | ☺ | ☺ | ☺ | 😐 |
|  | Saad (2009) | ☺ | ☹ | ☺ | ☺ | ☺ | ☺ | 😐 |
|  | Zhang (2023) | ☺ | ☹ | ☺ | ☺ | 😐 | ☺ | ☹ |
|  | Zheng Q. (2023) | ☺ | ☹ | ☺ | 😐 | ☺ | ☺ | 😐 |
| Other | Coelho (2013) | ☺ | ☹ | ☺ | 😐 | ☺ | ☺ | ☺ |
|  | Kim (2024) | ☺ | ☺ | ☺ | 😐 | ☺ | 😐 | ☺ |
|  | Livanelioglu (2015) | ☺ | ☹ | ☺ | ☺ | ☺ | ☺ | 😐 |
|  | Ovadia (2007) | ☺ | ☺ | ☺ | 😐 | ☺ | ☺ | 😐 |
|  | Salvia (2022) | ☺ | ☹ | ☺ | ☺ | ☺ | ☺ | 😐 |
|  | Solomito (2011) | ☹ | ☹ | ☺ | 😐 | ☺ | ☺ | 😐 |
|  | Takacs (2018) | ☺ | ☺ | ☺ | 😐 | ☺ | ☺ | 😐 |
|  | Wei (2023) | ☺ | ☺ | ☺ | ☺ | ☺ | 😐 | ☺ |
|  | Zabka (2015) | ☺ | ☹ | ☺ | ☺ | .?. | ☺ | 😐 |

## Accuracy and reliability of radiation-free scoliosis monitoring techniques

Surface topography

**Supplementary Table 5**: Overview of accuracy metrics of the ST category in terms of correlation with the reference standard and measurement difference to the reference standard.

| **STUDY** | **DIFFERENCE to X-RAY** | **CORRELATION with X-RAY** | **Progression detection** | **DEVICE** |
| --- | --- | --- | --- | --- |
| Adankon (2013) |  |  | Se=93%, Sp=80% | Self-developed |
| Berryman (2008) |  | r = 0.84 |  | Self-developed (ISIS2) |
| Bolzingern (2021) |  |  | Se=79%, Sp=63% | Biomod-L |
| De Korvin (2014) |  |  | Se=86%, Sp=50% | BIOMOD-L |
| Frerich (2012) | CA lumbar: MAD = 9.40°  CA thoracic: MAD = 7°  TKA: MAD = 10.16°  LLA: MAD = 8° | CA lumbar: r = 0.76  CA thoracic: r = 0.87  TKA: r = 0.80  LLA: r = 0.81 |  | DEIRS formetric 4D |
| Grunwald (2023) |  | r_s_ = 0.89 (Spearman) |  | Self-developed |
| Hong (2017) |  |  | Se=73%, Sp=44% | Self-developed |
| Knott (2016) | CA thoracic: MD = 5.8°  CA lumbar: MD = 8.8°  TKA: MD = 9.3°  LL: MD = 9.7° | CA thoracic: r = 0.73  CA lumbar: r = 0.49  TKA: r = 0.87  LL: r = 0.82 |  | DIERS formetric 4D |
| Komeili (2015) |  |  | Se=85.7% | Self-developed |
| Liang (2024) | CA lumber: MAD = 3.9° (SD = 3.4°)  CA thoracic: MAD = 5.2° (SD = 4.4°) |  |  | Self-developed (Kinect) |
| Meng (2023) | CA: MD = -0.86° | CA: r^2^ = 0.98 |  | Generic (Microsoft Azure Kinect) |
| Mishra (2020) | TKA: MAD = 3.61°  LLA: MAD = 4.09° | TKA: r = 0.85  LLA: r = 0.96 |  | DIERS statico 4D |
| Patel (2024) |  |  | Se=57.89%, Sp=84.21% | DIERS formetric 4D |
| Pino-Almero (2016) |  | CA vs. DHOPI: r = 0.72  CA vs. POTSI: r = 0.75 |  | Self-developed |
| Rothstock (2023) | CA thoracic: MAD = 8.39° (SD = 6.38°)  CA lumbar: MAD = 7.10° (SD = 6.46°) | CA thoracic: r^2^ = 0.74  Lumbar: r^ 2 = 0.48 |  | Generic (Structure Sensor, Occipital) |
| Schulte (2008) |  | Vertebral rotation: r = 0.5 |  | DIERS formetric 4D |
| Tabard-Fougère (2017) | CA: MD = 5.4° (SD = 4.5°) | CA: r = 0.70 |  | DIERS formetric 4D |
| Tabard-Fougère (2019) | TKA: MD = 6.9° (SD = 5.1°)  LLA: MD = 22.9° (SD = 8.9°) | TKA: r = 0.737  LLA: r = 0.605 |  | DIERS formetric 4D |
| Tabard-Fougère (2023) | CA: MD = 6.4° (SD = 4.7°) | CA: r = 0.89 |  | DIERS formetric 4D |
| Weiss (2013) |  | CA: r = 0.84 |  | DIERS formetric 4D |
| Yildirim (2021) |  |  |  | Generic (Artec EVA) |

*(MAD = mean absolute difference; MD = Mean Difference; SD = Standard Deviation; r = Pearson correlation coefficient; Se = sensitivity; Sp = specificity; CA = Cobb angle; TKA = thoracic kyphosis angle, LLA = lumbar lordosis angle).*

Ultrasonography

**Supplementary Table 8**: Overview of accuracy metrics of the US category. The accuracy is given in relation to radiographic (RG) measurements.

| **STUDY** | **US PARAM.** | **RG PARAM.** | **DIFFERENCE to X-RAY** | **CORRELATION with X-RAY** | **DEVICE** |
| --- | --- | --- | --- | --- | --- |
| Chen (2024) | COL | CA | MAD=2.91° (SD=2.36°) | r=0.89 | Generic US probe |
| Ferràs‑Tarragó (2019) | TPA | CA | MAD=2.91° (SD=2.36°) | r^2^=0.96 | Generic US probe |
| Huang (2024) | CA | CA | Proximal thoracic: MAD=1.50°; main thoracic: MAD=3.88°; thoracolumbar: MAD=4.17° |  | SCOLIOSCAN |
| Jiang (2022) | CA | CA |  | r=0.95 | SCOLIOSCAN |
| Lai (2023) | TPA | CA |  |  | SCOLIOSCAN |
| Lee TT. (2019) | SPA, COL (sagittal) | TKA, LLA |  | thoracic $r_{\mathrm{SPA}}^{2}$=0.64, $r_{\mathrm{COL}}^{2}$=0.70  lumbar: $r_{\mathrm{SPA}}^{2}$=0.66, $r_{\mathrm{COL}}^{2}$=0.57 | SCOLIOSCAN |
| Lee TT. (2021) | TPA | CA | Thoracic: MAD=3.0° (thoraco)lumbar: MAD= 2.8° | r^2^=0.88 | SCOLIOSCAN |
| Li DS. (2020) | SPI | CA (sagittal) |  | r=0.85 | Generic US probe |
| Li M. (2015) | SPA | CA |  | r=0.79 | Generic US probe |
| Lv (2020) | TP-SPA | CA |  | r=0.96 | Generic US probe |
| Wong (2019) | SPA | CA |  | r=0.816 | SCOLIOSCAN |
| Yang (2022) | TPA | CA | MAD=3.5 (SD=2.7°) thoracic: MAD=3.6 (SD=2.5°) lumbar: MAD=3.4 (SD=2.9°) | overall r^2^=0.815, thoracic r^2^=0.86; lumbar r^2^=0.79 | SCOLIOSCAN |
| Young (2015) | COL | CA | MAD= 2.6° to 4.1° |  | Generic US probe |
| Zeng (2024) | SPA | CA | MAD= 3.63° (SD = 2.6)° | r=0.72 | Generic US probe |
| Zheng R. (2015) | COL | CA | MAD=2.3° to 2.7° |  | Generic US probe |
| Zheng YP. (2016) | SPA | CA |  | r^2^=0.72 | SCOLIOSCAN |

*(Parameters (PARAM.): CA = Cobb angle; TKA = thoracic kyphosis angle; LLA = lumbar lordosis angle; TP(A) = transverse process (angle); SPA = spinous processes angle; SPI = sagittal projection image; COL = center of laminae. Metrics: MD = Mean Difference; MAD = mean absolute difference; r = Pearson correlation coefficient).*

Photogrammetry

**Supplementary Table 7**: Overview of accuracy metrics of the PG category in terms of correlation with the reference standard and measurement difference to the reference standard.

| **STUDY** | **DIFFERENCE to X-RAY** | **CORRELATION with X-RAY** | **Progression detection** | **DEVICE** |
| --- | --- | --- | --- | --- |
| Aroeira (2011) | MAD=4.1°  Thoracic: MAD=2.9°  Lumbar: MAD=5.1° |  |  | Generic camera |
| Leal (2019) |  | r=0.7 | Se=94.4%, Sp=86.7% | Generic camera |
| Saad (2008) |  | r=0.709 to 0.945 |  | Generic camera |
| Zhang (2023) |  |  | Se=63.33% (95% CI: 43.86%, 80.87%)  Sp=77.42% (95% CI: 58.90%-90.41%) | Smartphone |
| Zheng Q. (2023) |  | r=0.4 to max. 0.8 |  | Webcams |

*(Metrics: MD = Mean Difference; MAD = mean absolute difference; r = Pearson correlation coefficient, Se = sensitivity; Sp = specificity).*

Magnetic resonance imaging

**Supplementary Table 8**: Overview of accuracy metrics of the MRI category in terms of correlation with the reference standard and measurement difference to the reference standard.

| **STUDY** | **DIFFERENCE to X-RAY** | **CORRELATION with X-RAY** | **Progression detection** | **DEVICE** |
| --- | --- | --- | --- | --- |
| Diefenbach (2013) |  | r=0.901 |  | Upright positioning MRI |
| Lee MC (2013) |  | r=0.94 |  | Standard MRI, no axial loading |
| Roth (2024) | MD=3.3° (SD=9.4°) | *R*^2^=0.972 |  | Standard MRI, no axial loading, real-time |
| Schmitz (2001) | MD=-4.5° |  |  | Standard MRI, no axial loading |
| Wessberg (2006) | MD=-0.6° (SD=0.76°) | r=0.78 |  | Standard MRI, axial loading |

*(Metrics: MD = Mean Difference; MAD = mean absolute difference; r = Pearson correlation coefficient, Se = sensitivity; Sp = specificity).*

Other techniques

**Supplementary Table 9**: Overview of accuracy metrics of the “other” category in terms of correlation with the reference standard and measurement difference to the reference standard.

| **STUDY** | **DIFFERENCE to X-RAY** | **CORRELATION with X-RAY** | **Progression detection** | **DEVICE** |
| --- | --- | --- | --- | --- |
| Coelho (2013) |  | r=0.7 | Se=0.87, Sp=0.34 | Scoliometer |
| Kim (2024) |  |  | accuracy 92%, precision 91% | Inertial measurement units |
| Livanelioglu (2016) | MD=3.1° to 4.4° | r = 0.69 to 0.88 |  | Spinal Mouse |
| Ovadia (2007) | MAD=4 to 4.8° | r=0.86 |  | Ortelius800 |
| Salvia (2022) |  | R^2^=0.69 |  | Camera-based marker tracking system |
| Solomito (2011) |  | r=0.85 and 0.93 (not actual Cobb angles) |  | Camera-based marker tracking system |
| Takacs (2018) | MAD=4.98° (SD=3.73°) | R^2^=0.7 |  | ZEBRIS spine |
| Wei (2023) |  | Thoracic: r=0.6 lumbar: r=0.5 |  | SpineScan3D (digital Scoliometer) |
| Zabka (2015) | MAD=7° (SD=5.68°) |  |  | Ortelius800 |

*(Metrics: MD = Mean Difference; MAD = mean absolute difference; r = Pearson correlation coefficient, Se = sensitivity; Sp = specificity).*
